# Supplementary figures and images for: Mutation rate dynamics reflect ecological change in an emerging zoonotic pathogen
Source: PLoS Genet. 2021 Nov 8;17(11):e1009864. doi: 10.1371/journal.pgen.1009864 (PMC8601623; doi:10.1371/journal.pgen.1009864)

(a) Population structure

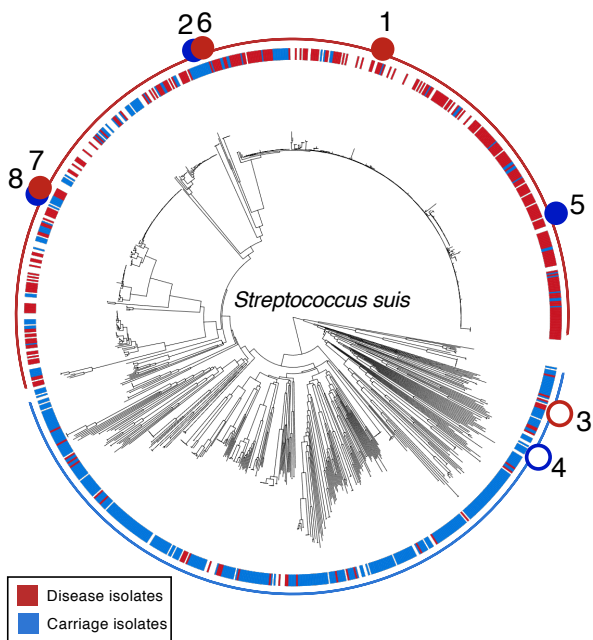

(b) Disease association

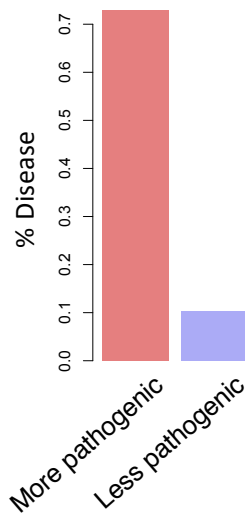

(c) Genome size

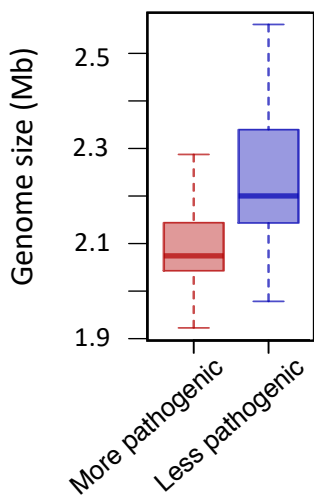

(d) Core genome GC-content

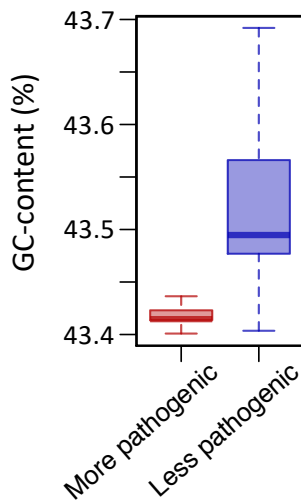

Supplement: S1 Fig — (a) A core genome phylogeny of 962 isolates of S. suis [12]. Individual disease (red) and carriage (blue) isolates are indicated in the inner strip. The more pathogenic clade is indicated by a red outer ring, and the less pathogenic clade by a blue outer ring. The locations of each strain in our two MA experiments are indicated on this strip (Table 1). (b) The proportion of isolates in each clade that are associated with disease (excluding isolates for which disease-association is unknown). (c) A box plot of genome sizes of isolates from each clade. (d) A box plot of the core genome GC-content of isolates from each clade. (PDF) [file pgen.1009864.s001.pdf]

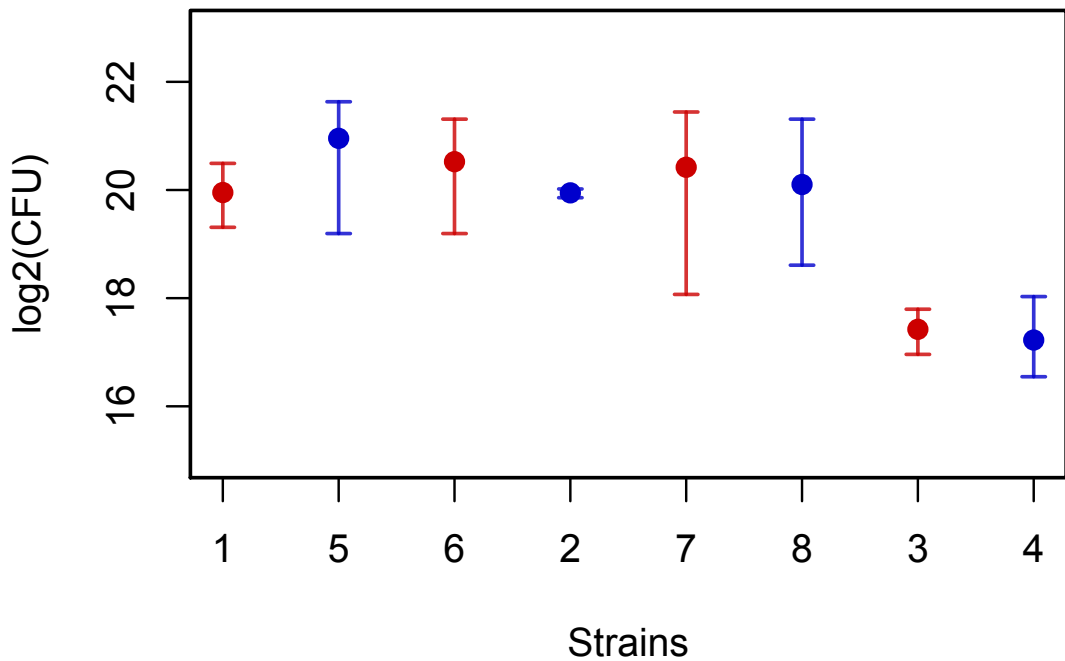

Supplement: S2 Fig — Points represent mean estimates of the number of colony forming units (CFU) present after 24 hours of growth from a single CFU for each ancestral strain, on a log2 scale. Bars show the range of values returned across biological replicates (at least 3 biological replicates of each strain). Disease strains are shown in red and carriage strains in blue. Log2(CFU) after 24 hours of growth gives an estimate of the number of generations over that period. We find no evidence of a difference in generation time between disease and carriage strains, but the two strains from the less pathogenic clade (strains 3 and 4) have a longer generation time than the six strains from the more pathogenic clade (strains 1, 2, 5, 6, 7 and 8). (PDF) [file pgen.1009864.s002.pdf]

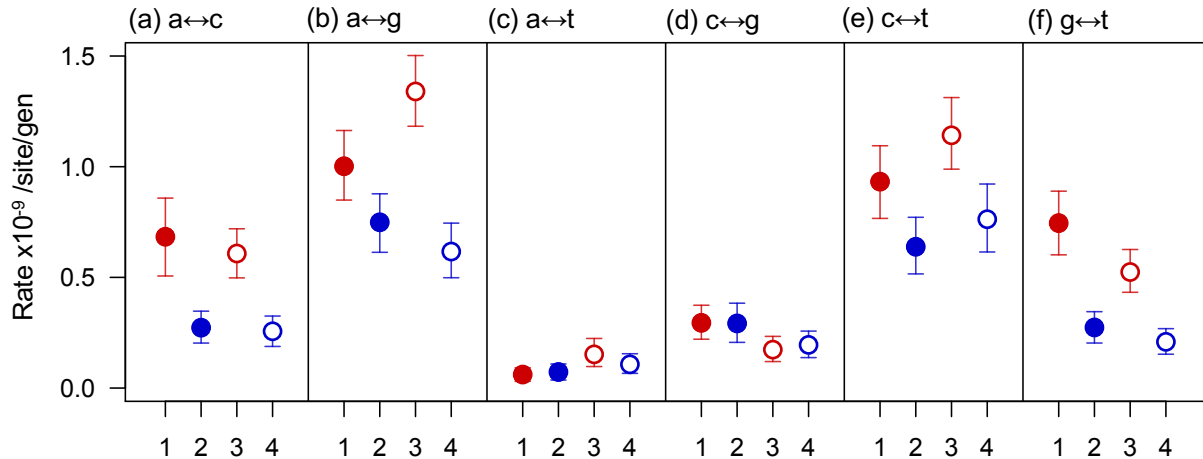

Supplement: S3 Fig — Points represent mean values across 50 replicate lines, and bars represent 95% confidence intervals estimated from bootstrapping across lines. Numbers relate to Table 1; disease strains are shown in red and carriage in blue, strains from the more pathogenic clade are shown as filled shapes and strains from the less pathogenic clade as empty shapes. (PDF) [file pgen.1009864.s003.pdf]

(a)

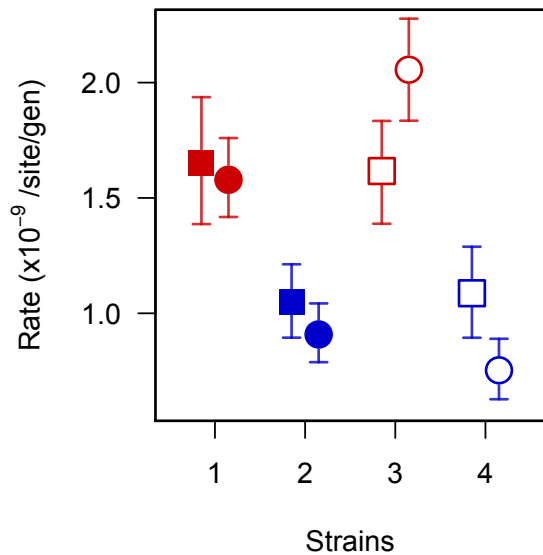

(b)

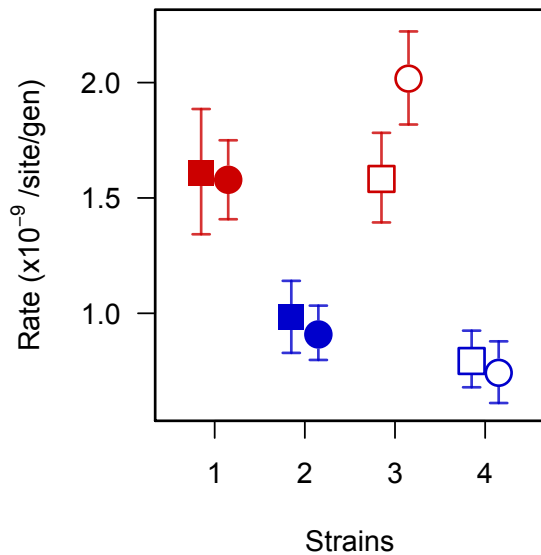

Supplement: S4 Fig — Comparison of mutation rates across core and accessory genes in the 200-day MA experiment, with (a) and without clustered mutations (b). Estimates of rates of single-base mutation rates across accessory (squares) and core (circles) genes. Points represent mean values across 50 replicate lines, and bars represent 95% confidence intervals estimated from bootstrapping across lines. Numbers relate to Table 1; disease strains are shown in red and carriage in blue, strains from the more pathogenic clade are shown as filled shapes and strains from the less pathogenic clade as empty shapes. (PDF) [file pgen.1009864.s004.pdf]

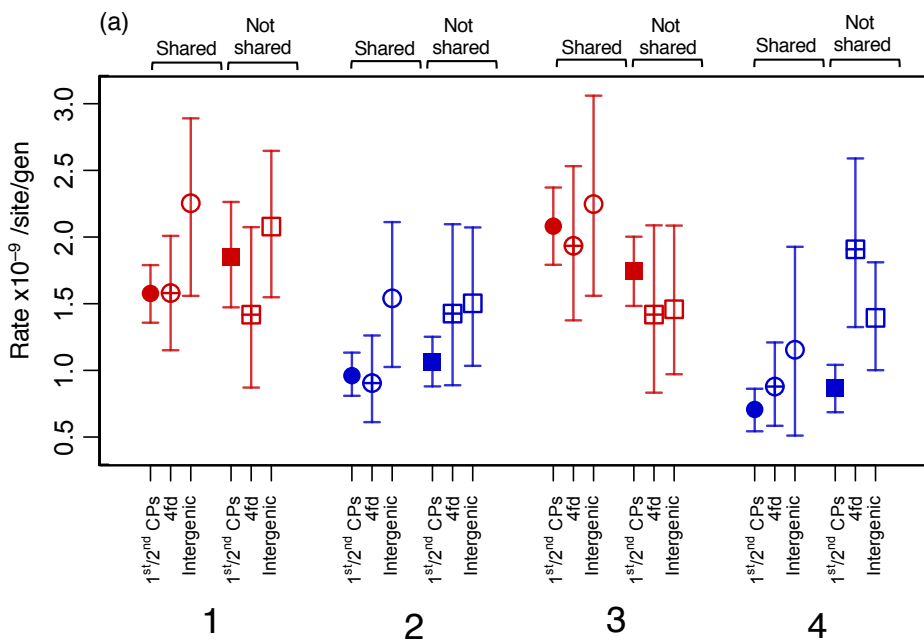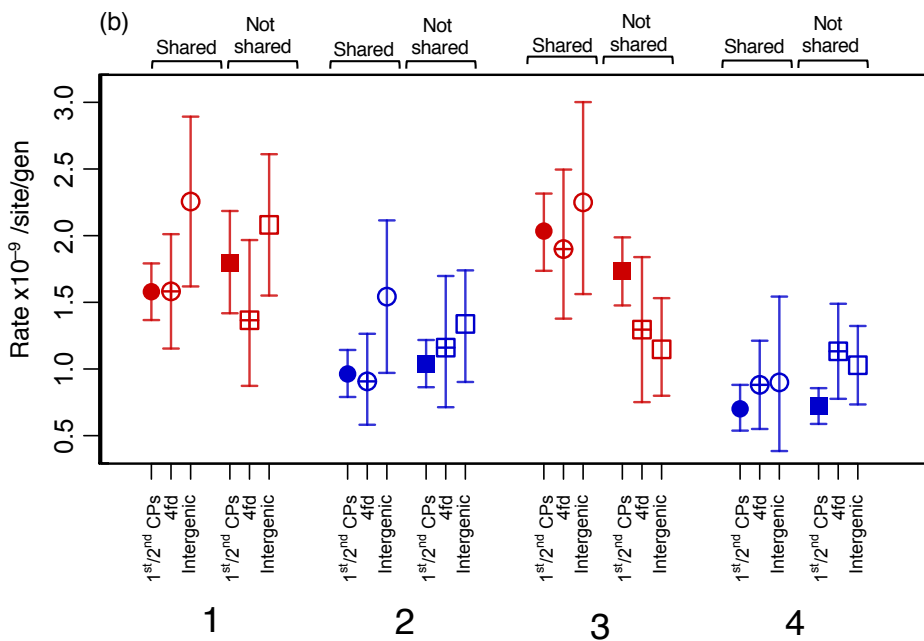

Supplement: S5 Fig — Comparison of mutation rates in the 200-day MA experiment across different categories of sites, with (a) and without clustered mutations (b). Both figures show rates for 1st/2nd codon positions, four-fold degenerate sites, and intergenic sites, for both regions that are shared across all four strains and regions that are not shared. Shared intergenic sites were defined as intergenic regions between genes that were syntenic across all four strains. Estimates based only on genes that are shared across all four strains (core) are shown as circles, and estimates based only on genes that are not shared across all four strains (accessory) are shown as squares. Disease strains are shown in red and carriage strains in blue. All points represent mean values across 50 replicate lines, and bars represent 95% confidence intervals estimated from bootstrapping across lines. (PDF) [file pgen.1009864.s005.pdf]

**1**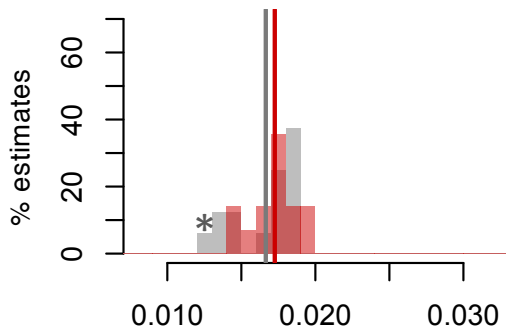**2**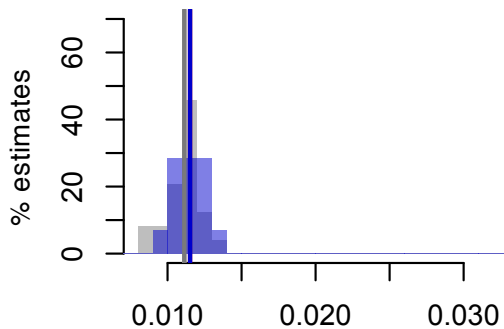**3**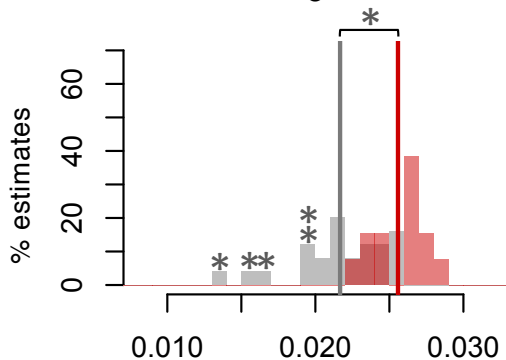**4**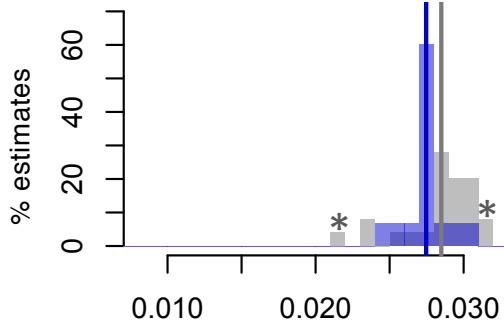

Max. growth rate (change in OD per min)

Supplement: S6 Fig — Histograms of maximum growth rates (change in optical density (OD) per min) for evolved lines (mean values across 3 biological replicates; shown in grey) and repeated measurements of the ancestral line (red for disease and blue for carriage). Estimates were attempted for a random sample of 25 evolved lines for each strain, however insufficient overnight growth meant that we could not obtain accurate maximum growth rate estimates for 9/25 evolved lines of strain 1 and 1/25 evolved lines of strain 2. Vertical lines represent mean values across repeated measurements of the ancestral strain (red/blue), and across evolved lines (grey). For strain 3 there is evidence of a net decline in maximum growth rate in the evolved lines (Welch’s t-test, p = 2.9 x 10^−5^, indicated by * above brackets). For the three other strains there is no evidence of a net change in the maximum growth rate. Estimates of maximum growth rate for 7 individual evolved lines were significantly lower than estimates for the ancestral strain, and 1 significantly higher (Welch’s t-test, p < 0.05 after Bonferroni correction for multiple testing, indicated by * above bars). (PDF) [file pgen.1009864.s006.pdf]

(a)

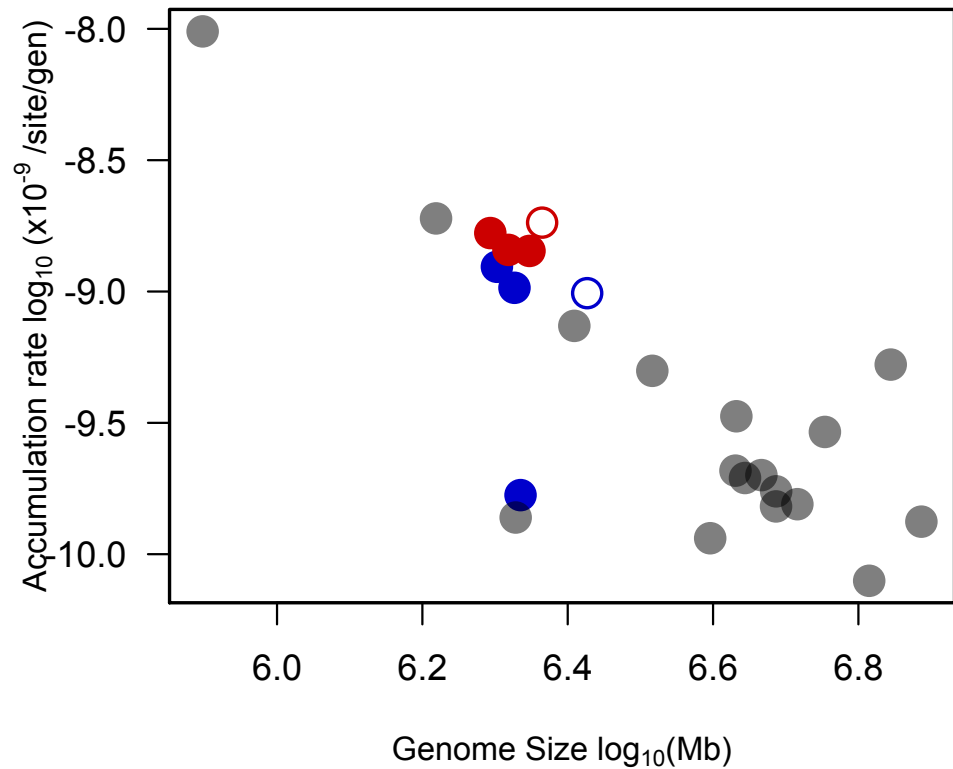

(b)

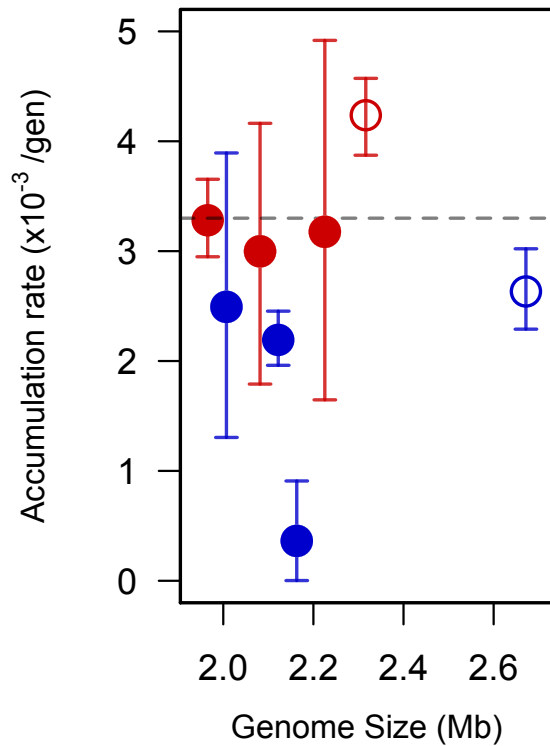

Supplement: S8 Fig — (a) Mutation rate estimates for S. suis in the context of those of other species, and (b) a comparison of mutation rate estimates per genome. (a) Mutation rate estimates against genome size, for S. suis, Mesoplasma florum, Helicobacter pylori, Thermus thermophilus, Staphylococcus epidermidis, Deinococcus radiodurans, Vibrio cholerae, Vibrio fischeri, Bacillus subtilis, Mycobacterium tuberculosis, Escherichia coli, Salmonella typhimurium, Salmonella enterica, Teredinibacter turnerae, Agrobacterium tumefaciens, Pseudomonas aeruginosa, Mycobacterium smegmatis and Burkholderia cenocepacia [1, 56]. S. suis disease isolates are shown in red and carriage isolates in blue, with closed circles representing isolates from the more pathogenic clade and open circles isolates from a less pathogenic clade, other species are shown in grey. In (b) the dashed line indicates the value of the constant mutation rate identified by Drake in his original study of the relationship between mutation rate and genome size [16]. (PDF) [file pgen.1009864.s008.pdf]

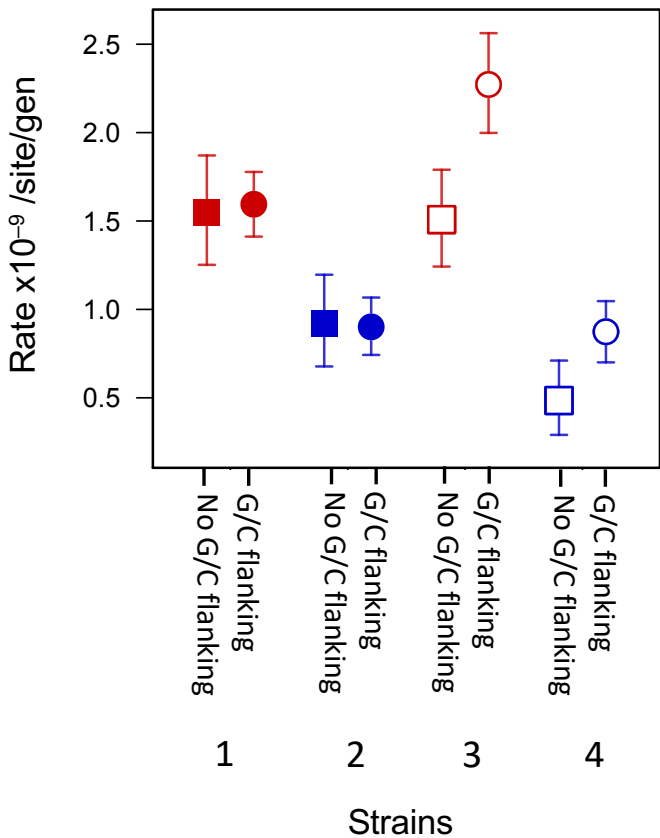

Supplement: S9 Fig — Estimates of mutation rates for sites that have at least one G/C flanking site, and estimates of rates for sites that have no G/C flanking site for each of the four strains from the longer experiment. Points represent mean values across 50 lines, and bars represent 95% confidence intervals from bootstrapping across lines. (PDF) [file pgen.1009864.s009.pdf]

**(a)**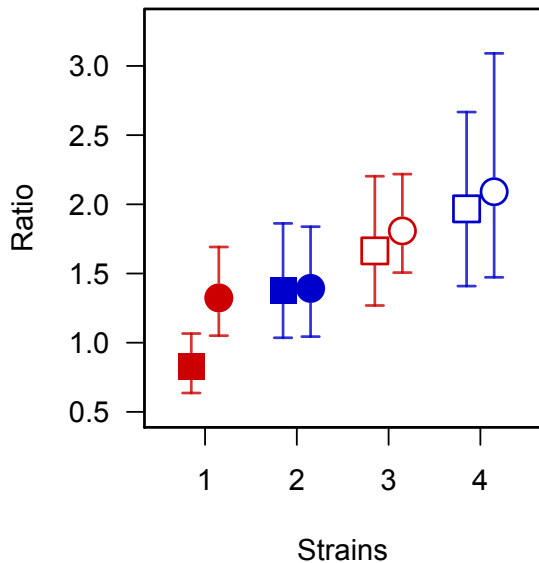**(b)**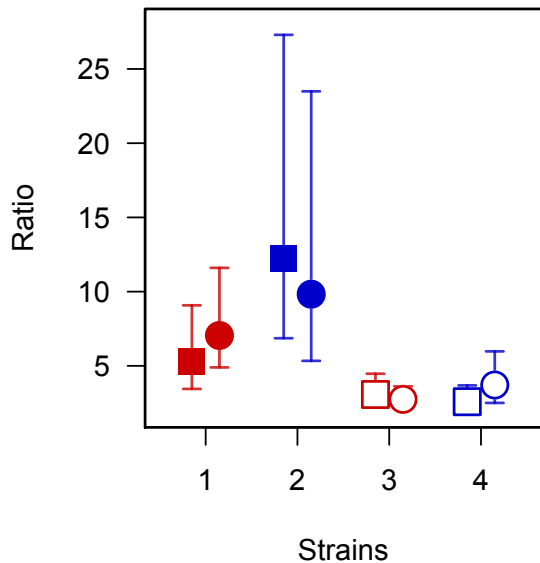

Supplement: S10 Fig — (a) The ratio of transitions to transversions for shared (circles) and non-shared (squares) regions of the genome. (b) The ratio of G/C to AT transitions to A/T to G/C transitions for non-shared (squares) and shared (circles) regions of the genome. Disease-associated strains are coloured in red circles and carriage strains in blue, with filled shapes representing isolates from the more pathogenic group and empty shapes isolates from the less pathogenic group. All points represent mean values across 50 lines, and bars represent 95% confidence intervals estimated by bootstrapping. (PDF) [file pgen.1009864.s010.pdf]

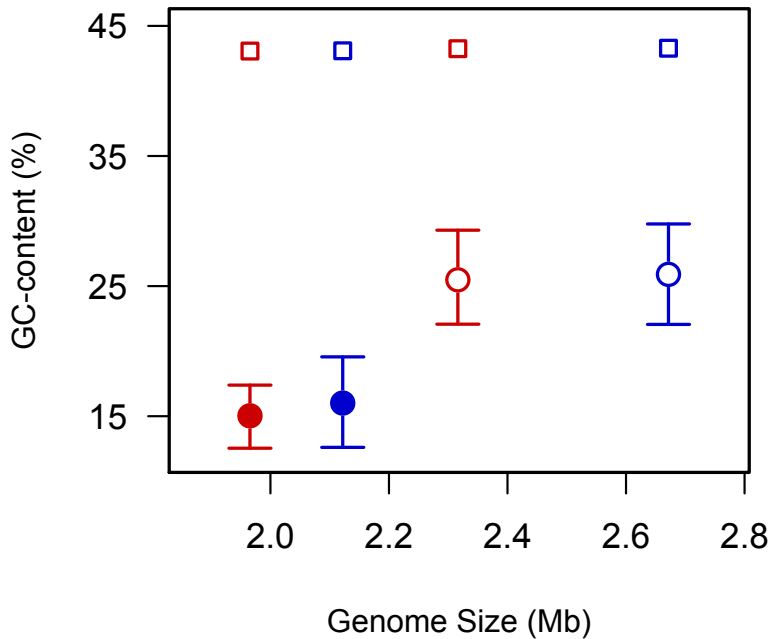

Supplement: S11 Fig — Equilibrium GC-content estimates, calculated from the rates of A/T to G/C mutation and G/C to A/T mutation (circles) and actual genome-wide GC-content for the four strains (squares). Disease-associated strains are coloured in red and carriage strains in blue, with filled shapes representing isolates from the more pathogenic group and empty shapes isolates from the less pathogenic group. All points represent mean values across 50 lines, and bars represent 95% confidence intervals estimated by bootstrapping. (PDF) [file pgen.1009864.s011.pdf]

**(a)**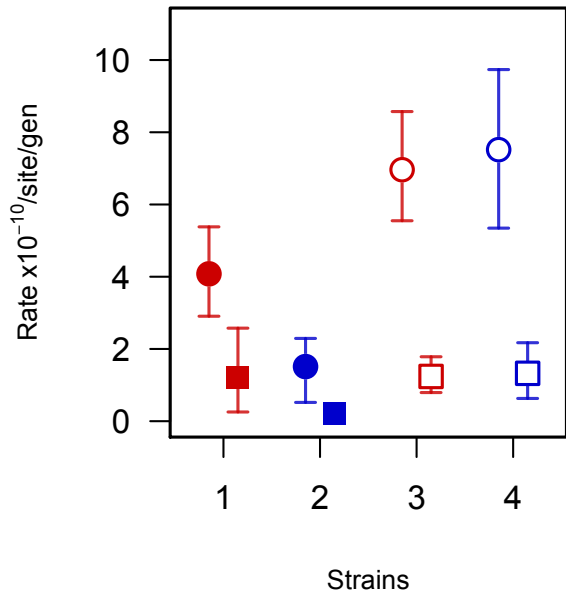**(b)**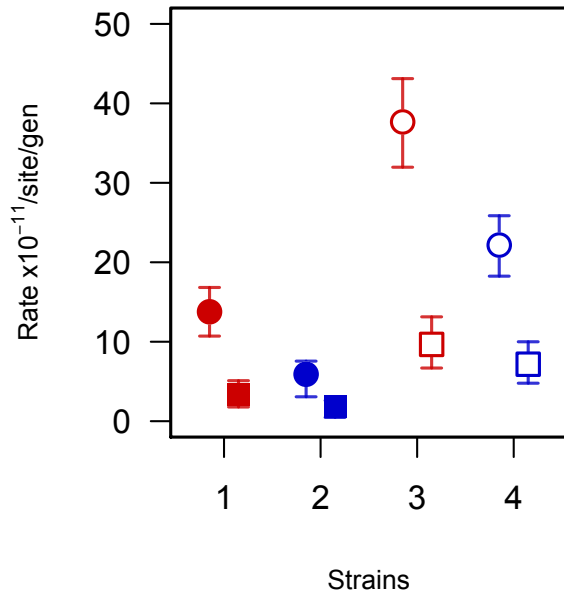

Supplement: S12 Fig — (a) The rates of loss/gain of nucleotide bases through short deletion (circles) and insertion (square) events, and (b) the rates of short deletion (circle) and insertion (squares) events. All points represent mean values across 50 lines, and bars represent 95% confidence intervals estimated by bootstrapping. (PDF) [file pgen.1009864.s012.pdf]

(a)

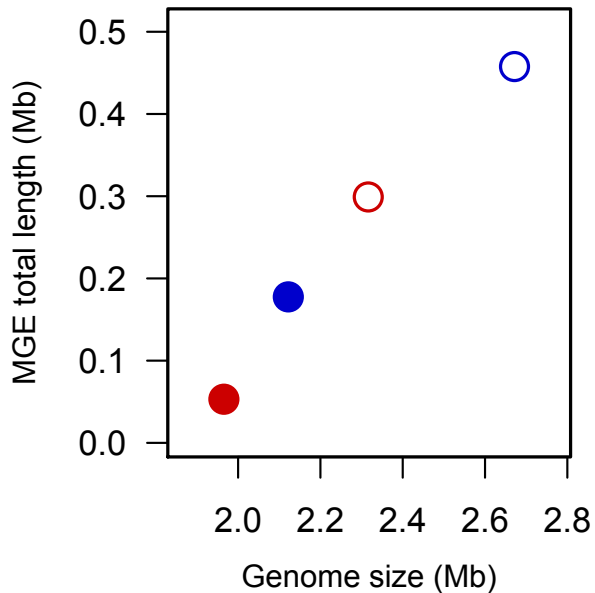

(b)

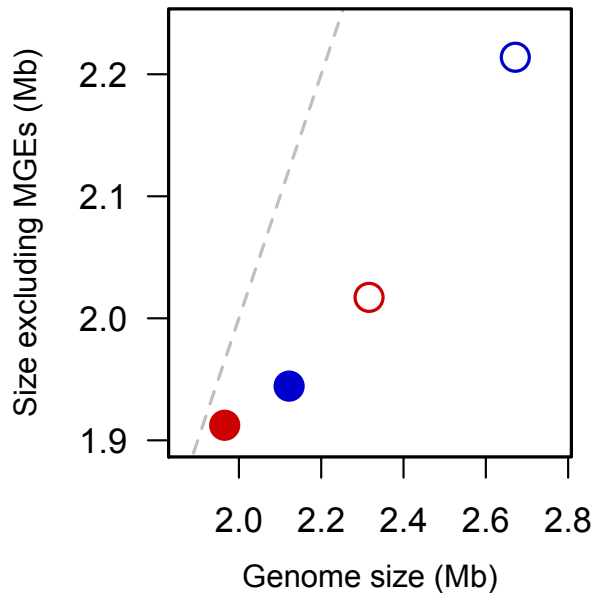

Supplement: S13 Fig — Figures show the relationship between the total length of mobile genetic elements in each strain and total genome size (a), and the relationship between the length of the genome excluding mobile genetic elements and total genome size (b). (PDF) [file pgen.1009864.s013.pdf]
