# Supplementary material for: Mutation rate dynamics reflect ecological change in an emerging zoonotic pathogen
Source: PLoS Genet. 2021 Nov 8;17(11):e1009864. doi: 10.1371/journal.pgen.1009864 (PMC8601623; doi:10.1371/journal.pgen.1009864)
Supplement: S7 Fig — Maximum growth rate (change in optical density (OD) per min) plotted against the number of single-base substitutions accumulated over the course of the experiment for evolved lines of each strain. Growth rate estimates were attempted for a random sample of 25 evolved lines of each strain, however insufficient overnight growth meant that we could not obtain accurate maximum growth rate estimates for 9/25 lines of strain 1 and 1/25 lines of strain 2. The estimates of maximum growth rates shown are mean values across 3 biological replicates. Spearman’s ρ and p-values for correlation tests are reported for each strain. There is no evidence of a significant correlation between maximum growth rate and the number of mutations accumulated for any of our four strains. However, the 9 lines of strain 1 that had insufficient overnight growth had more single-base mutations (average of 15.4) than 16 lines that had sufficient overnight growth (average of 11.4) (Welch’s t-test, p = 0.04). Similarly, the line of strain 2 than had insufficient overnight growth had a high number of single-base mutations (n = 13) compared to the other lines of this strain included in the experiment. (PDF) [file pgen.1009864.s007.pdf]

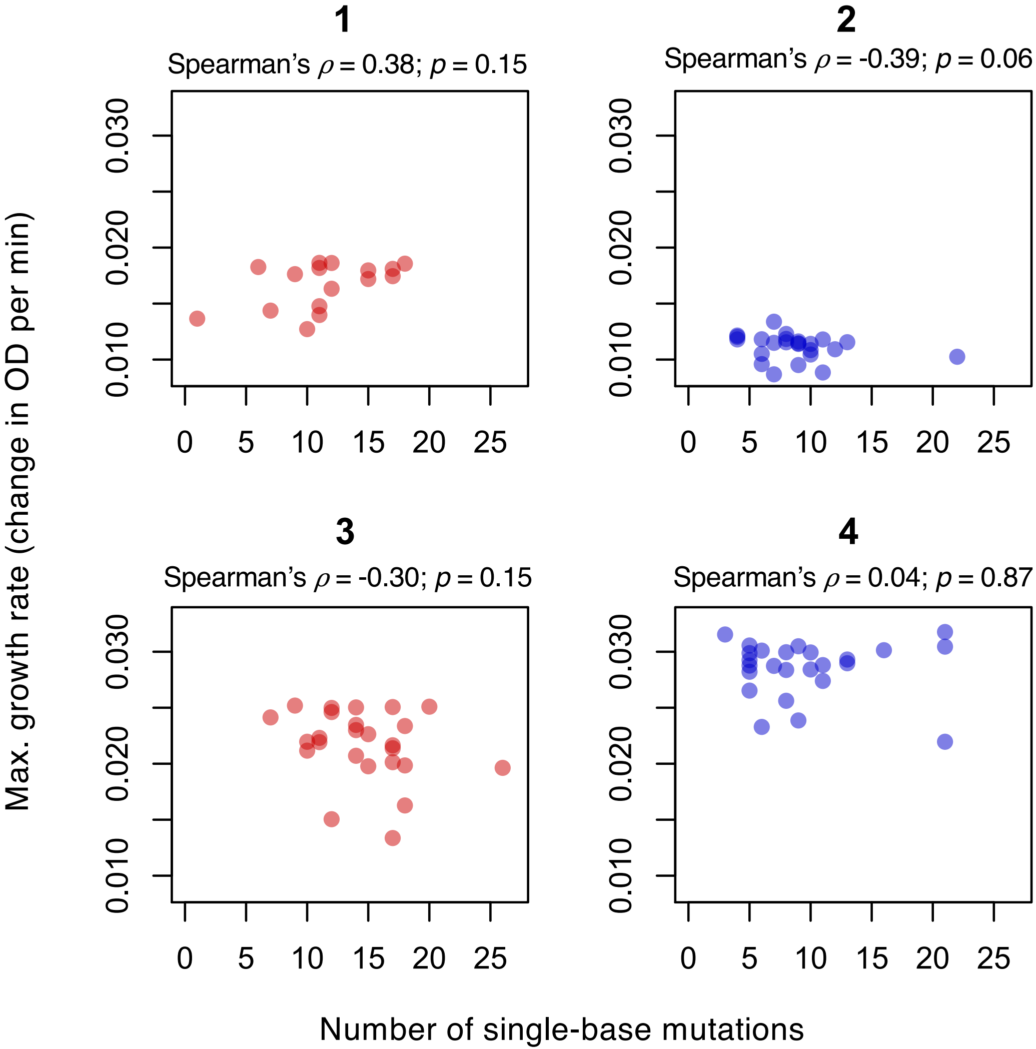
1
Spearman's ρ = 0.38; p = 0.15
2
Spearman's ρ = -0.39; p = 0.06
3
Spearman's ρ = -0.30; p = 0.15
4
Spearman's ρ = 0.04; p = 0.87
0.010
0.020
0.030
0
5
10
15
20
25
Max. growth rate (change in OD per min)
Number of single-base mutations
