# Supplementary material for: Mutation rate dynamics reflect ecological change in an emerging zoonotic pathogen
Source: PLoS Genet. 2021 Nov 8;17(11):e1009864. doi: 10.1371/journal.pgen.1009864 (PMC8601623; doi:10.1371/journal.pgen.1009864)
Supplement: S1 Table — (DOCX) [file pgen.1009864.s014.docx]

**Table S1. Description of the eight strains used in both MA experiments.**

| **Disease- association** | Disease | Carriage | Disease | Carriage | Disease | Carriage | Disease | Carriage |
| --- | --- | --- | --- | --- | --- | --- | --- | --- |
| **Genetic background** | More pathogenic clade | | | | | | Less pathogenic clade | |
| **Strain** | 1 | 5 | 6 | 2 | 7 | 8 | 3 | 4 |
| **Country of origin** | Canada | Spain | The Netherlands | Canada | Denmark | Canada | Canada | UK |
| **Year of sampling** | 2014 | 2015 | 2017 | 2016 | 2018 | 2016 | 2009 | 2013 |
| **Cluster [12]** | 1 | | 12 | | 3 | | 10 | |
| **No. lines sequenced** | 50 | 13 | 12 | 50 | 13 | 11 | 50 | 50 |
| **Genome size (Mb)** | 1.97 | 2.00 | 2.11 | 2.12 | 2.22 | 2.10 | 2.32 | 2.67 |
| **No. genes** | 1952 | 1964 | 2055 | 2120 | 2241 | 2144 | 2344 | 2712 |
| **Length coding (Mb)** | 1.74 | 1.76 | 1.83 | 1.89 | 1.96 | 1.90 | 2.09 | 2.36 |
| **%GC (core genome)** | 0.4309 | 0.4309 | 0.4309 | 0.4310 | 0.4311 | 0.4312 | 0.4330 | 0.4330 |
| **Generation time (mins)** | 70 | 67 | 68 | 72 | 67 | 68 | 81 | 80 |
| **Previous strain names and references** | 1619952 (Hadjirin et al. 2021) [1] | - | - | DB1V3-4A  (Hadjirin et al. 2021) [1] | - | MZ1B3-4E  (Hadjirin et al. 2021) [1] | 1191316 (Hadjirin et al. 2021) [1] | 684-21B (Wileman et al. 2019) [2] |
| **Long-read data** | ENA: SAMEA5610089 (PRJEB21775) | MicrobesNG: 5304491C-278D-4B29-AA2A-D67A13DEF6B5 | MicrobesNG: 5304491C-278D-4B29-AA2A-D67A13DEF6B5 | ENA: SAMEA5610090 (PRJEB21775) | MicrobesNG: 08410916-9F24-47E7-9412-5AC9E3ADAD7B | MicrobesNG: 08410916-9F24-47E7-9412-5AC9E3ADAD7B | ENA: SAMEA5610088 (PRJEB21775) | ENA: SAMEA5610091 (PRJEB21775) |
| **Short-read data** | (Hadjirin et al. 2021) [1] | MicrobesNG: 5304491C-278D-4B29-AA2A-D67A13DEF6B5 | MicrobesNG: 5304491C-278D-4B29-AA2A-D67A13DEF6B5 | (Hadjirin et al. 2021) [1] | MicrobesNG: 08410916-9F24-47E7-9412-5AC9E3ADAD7B | MicrobesNG: 08410916-9F24-47E7-9412-5AC9E3ADAD7B | (Hadjirin et al. 2021) [1] | (Wileman et al. 2019) [2] |
| **Assembly NCBI Accession** | CP085088 | JAJBSM000000000 | CP085086 | CP085087 | JAJDOQ000000000 | CP085085 | JAJBSN000000000 | CP084908 |

**References:**

1. Hadjirin N.F., Miller E.L., Murray G.G.R., Yen P.L.K., Phuc H.D., Wileman T.M., Hernandez-Garcia J., Williamson S.M., Parkhill J., Maskell D.J., Zhou R., Fittipaldi N., Gottschalk M., Tucker A.W., Hoa N.T., Welch J.J., Weinert L.A. Large-scale genomic analysis of antimicrobial resistance in the zoonotic pathogen *Streptococcus suis*. BMC Biol. 2021;19:1–17.

2. Wileman T.M., Weinert L.A., Howell K.J., Wang J., Peters S.E., Williamson S.M., Wells J.M., Langford P.R., Rycroft A.N., Wren B.W., Maskell D.J., Tucker A.W.. Pathotyping the Zoonotic Pathogen *Streptococcus suis*: Novel genetic markers to differentiate invasive disease-associated isolates from non-disease-associated isolates from England and Wales. J Clin Microbiol. 2019; 57(7):e01712-18.
