# Supplementary material for: Mutation rate dynamics reflect ecological change in an emerging zoonotic pathogen
Source: PLoS Genet. 2021 Nov 8;17(11):e1009864. doi: 10.1371/journal.pgen.1009864 (PMC8601623; doi:10.1371/journal.pgen.1009864)
Supplement: S4 Table — Counts were divided into four categories for each strain, with each category having an expected frequency >5 under a Poisson distribution given our estimates of the mean rate. (DOCX) [file pgen.1009864.s017.docx]

**Table S4. Results of Chi-Squared test for the goodness of fit of a Poisson distribution to mutation rates of single-base substitutions in the 200-day MA experiment.** Counts were divided into four categories for each strain, with each category having an expected frequency >5 under a Poisson distribution given our estimates of the mean rate.

| **Strain** | **Single-base substitutions** | | |
| --- | --- | --- | --- |
|  | **𝞦^2^** | ***df*** | ***p*** |
| **1** | **4.21** | **3** | **0.24** |
| **2** | **1.49** | **3** | **0.71** |
| **3** | **1.48** | **3** | **0.69** |
| **4** | **6.10** | **3** | **0.11** |
