# Supplementary material for: Mutation rate dynamics reflect ecological change in an emerging zoonotic pathogen
Source: PLoS Genet. 2021 Nov 8;17(11):e1009864. doi: 10.1371/journal.pgen.1009864 (PMC8601623; doi:10.1371/journal.pgen.1009864)
Supplement: S6 Table — 5 lines of each strain in the 200-day MA experiment were sequenced at the mid-point of the experiment to establish whether faster rates were transitory. Lines were selected that had higher than average rates from each strain. We found no evidence of a difference in rate over the first and second half of the experiment. (DOCX) [file pgen.1009864.s019.docx]

**Table S6. Comparison of numbers of single-base mutations observed at day 100 and day 200 in the 200-day experiment.** 5 lines of each strain in the 200-day MA experiment were sequenced at the mid-point of the experiment to establish whether faster rates were transitory. Lines were selected that had higher than average rates from each strain. We found no evidence of a difference in rate over the first and second half of the experiment.

| **Strain** | **Number of SNPs** | |
| --- | --- | --- |
|  | **200 days** | **100 days** |
| **1** | 26 | 12 |
|  | 22 | 12 |
|  | 12 | 6 |
|  | 17 | 9 |
|  | 24 | 14 |
| **2** | 22 | 10 |
|  | 16 | 6 |
|  | 16 | 9 |
|  | 14 | 1 |
|  | 10 | 10 |
| **3** | 26 | 10 |
|  | 22 | 10 |
|  | 21 | 9 |
|  | 21 | 10 |
|  | 17 | 6 |
| **4** | 21 | 10 |
|  | 21 | 13 |
|  | 21 | 15 |
|  | 20 | 7 |
|  | 18 | 5 |
