# Supplementary material for: Mutation rate dynamics reflect ecological change in an emerging zoonotic pathogen
Source: PLoS Genet. 2021 Nov 8;17(11):e1009864. doi: 10.1371/journal.pgen.1009864 (PMC8601623; doi:10.1371/journal.pgen.1009864)
Supplement: S7 Table — The proportion of nucleotide bases that differ between pairs of strains in the MA experiments based on an alignment of shared genes generated by Panaroo. Closely related disease/carriage pairs are highlighted. (DOCX) [file pgen.1009864.s020.docx]

**Table S7. Core genome pairwise nucleotide distances between the eight ancestral strains used in the MA experiments.** The proportion of nucleotide bases that differ between pairs of strains in the MA experiments based on an alignment of shared genes generated by Panaroo. Closely related disease/carriage pairs are highlighted.

|  | **1**  (disease) | **5**  (carriage) | **6**  (disease) | **2**  (carriage) | **8**  (disease) | **7**  (carriage) | **3**  (disease) | **4**  (carriage) |
| --- | --- | --- | --- | --- | --- | --- | --- | --- |
| **1** |  | 1.20x10^-4^ | 1.78x10^-2^ | 1.78x10^-2^ | 3.02x10^-2^ | 3.03x10^-2^ | 5.49x10^-2^ | 5.62x10^-2^ |
| **5** |  |  | 1.78x10^-2^ | 1.78x10^-2^ | 3.02x10^-2^ | 3.02x10^-2^ | 5.49x10^-2^ | 5.62x10^-2^ |
| **6** |  |  |  | 7.39x10^-5^ | 2.92x10^-2^ | 2.91x10^-2^ | 5.45x10^-2^ | 5.57x10^-2^ |
| **2** |  |  |  |  | 2.92x10-^2^ | 2.91x10^-2^ | 5.45x10^-2^ | 5.57x10^-2^ |
| **8** |  |  |  |  |  | 1.06x10^-3^ | 5.53x10^-2^ | 5.61x10^-2^ |
| **7** |  |  |  |  |  |  | 5.53x10^-2^ | 5.62x10^-2^ |
| **3** |  |  |  |  |  |  |  | 4.85x10^-2^ |
| **4** |  |  |  |  |  |  |  |  |
