# Supplementary material for: Mutation rate dynamics reflect ecological change in an emerging zoonotic pathogen
Source: PLoS Genet. 2021 Nov 8;17(11):e1009864. doi: 10.1371/journal.pgen.1009864 (PMC8601623; doi:10.1371/journal.pgen.1009864)
Supplement: S11 Table — The lengths of the regions identified by IslandViewer for each strain. (DOCX) [file pgen.1009864.s024.docx]

**Table S11. The size of regions identified as mobile genetic elements for each strain in the 200-day MA experiment.** The lengths of the regions identified by IslandViewer for each strain.

| **Strain** | **Number of genes in mobile elements** | **Length of mobile elements, bp (%)** |
| --- | --- | --- |
| Small-genome pathogen | 58 | 53,085 (2.7 %) |
| Small-genome carriage | 205 | 177,561 (8.3 %) |
| Large-genome pathogen | 271 | 299,087 (12.9 %) |
| Large-genome carriage | 505 | 457,681 (17.1 %) |
