# Supplementary material for: Mutation rate dynamics reflect ecological change in an emerging zoonotic pathogen
Source: PLoS Genet. 2021 Nov 8;17(11):e1009864. doi: 10.1371/journal.pgen.1009864 (PMC8601623; doi:10.1371/journal.pgen.1009864)
Supplement: S15 Table — (DOCX) [file pgen.1009864.s028.docx]

**Table S15. Oligonucleotide primers (Sigma-Aldrich) and conditions used for Multiplex PCR strain identification.**

| **Primers** | | | | | | **Thermocycling conditions** | **PCR reagents per sample (10 µl)** |
| --- | --- | --- | --- | --- | --- | --- | --- |
| **Name** | **Sequence (5’-3’)** | **Product size (bp)** | | | |  |  |
|  |  | **Strain 1** | **Strain 2** | **Strain 3** | **Strain 4** |  |  |
| MA-F2 | ATACTCAATGAAA  ATCAGAATCAGA | 570 | 570 | 730 | 840 | 95ºC - 10min  x1 cycle  95ºC - 30s  53ºC - 30s  72ºC - 1min  x 30 cycles    72ºC 5min  x1 cycle | **1 µl** DNA template  **5.3375 µl** MilliQ H2O  **2 µl** MyTaq Red Reaction Buffer  **0.375 µl** of the primer  **0.0625 µl** MyTaq DNA polymerase  **0.1 µl** DMSO |
| MA-R2 | TCGGTCAAAT  GGTTTGTCG | 570 | 570 | 730 | 840 |  |  |
| MA-F5 | ATTTGATGACT  GAAAAGCTCCT | - | 234 | 284 | - |  |  |
| MA-R5 | GGTTTGAAGTT  ATAATAAAAAACAA | 284 | 234 | 284 | 234 |  |  |
| SsuisF | CTGTAAACCAAT CCATCTTGA | 853 | 853 | 853 | 853 |  |  |
| SsuisR | CTCATTTCAAGG GCAGATAC | 853 | 853 | 853 | 853 |  |  |
