# Supplementary material for: Mutation rate dynamics reflect ecological change in an emerging zoonotic pathogen
Source: PLoS Genet. 2021 Nov 8;17(11):e1009864. doi: 10.1371/journal.pgen.1009864 (PMC8601623; doi:10.1371/journal.pgen.1009864)
Supplement: Attachment — Submitted filename: MA - Response to Reviewers.docx [file pgen.1009864.s030.docx]

Responses to Reviewer **#**1

1.0 We would like to thank the reviewer for their positive assessment of the rigor of our study, and their helpful comments. We have addressed each of their concerns as detailed below. Page and line numbers refer to the pdf document with highlighted changes.

**Reviewer #1:** The authors quantitatively measure mutation rate in 4 pairs of S. suis strains. One member of each pair is pathogenic, while the other is commensal. In each of the 4 pairs, the pathogenic clone has a higher mutation rate than the commensal clone. From these findings, the authors suggest that "ecology is a stronger correlate of mutation rate than genome size over these timescales, and that transitions to invasive disease are consistently accompanied by rapid increases in mutation rate".

I believe the rigor of the authors' protocol for measuring mutation rates. However, my opinion is that two aspects of the authors' claims are too strong with respect to what their data show. I suggest that the authors either temper these claims, or provide more conclusive evidence to support them.

These claims are: 1) recent pathogenic strains have higher mutation rates than commensal carriage strains of S. suis. 2) transitions to invasive disease are consistently accompanied by rapid increases in mutation rate.

Regarding claim 1. While the authors' data is suggestive, I do not think we can draw a definitive conclusion about the association between ecology and mutation rate in S. suis. The small sample size (4 pairs of clones-- due to phylogenetic correlations between all clones, I see this as 4 independent data points) prevents a strong conclusion in this regard. As a quick statistical sanity check, the odds of the pathogen strain having a higher mutation rate than the commensal strain in all four pairs by chance, is the same as the odds of flipping a coin 4 times and getting 4 heads (p = 0.065). Hence, I am not convinced that the 4 pairs of recent pathogen/commensal strains is sufficient to claim a strong association between ecology and mutation rate in S. suis. I would prefer to see more pairs so that the authors can make definitive statistical claims for or against their hypothesis. Alternatively, the authors could weaken their conclusions, by stating that these data favor their hypothesis, but that more data (more pathogen/commensal pairs-- not more MA lines from the existing pairs) are needed to definitively test it.

1.1 We appreciate the reviewer's concern about the power of our study. Mutation accumulation experiments are extremely labour intensive, and this limited the number of strains we were able to obtain mutation rate estimates for. The reviewer is correct that when you use a sign test, the probability that the elevation in rates in disease isolates we observe happened by chance is 0.065. In our manuscript we report the results of a paired t-test, which indicates that the probability that the difference we observe between disease and carriage isolates arose by chance is 0.035. As we have no reason to expect non-normality in the distribution of differences in mutation rates, and we see no evidence of non-normality in our data (a Shapiro-Wilk test shows no significant deviation from normality, *p* = 0.28), we consider that our use of a t-test is justifiable. Moreover, unpaired tests of a difference between disease and carriage mutation rates (both parametric t-test and non-parametric Mann-Whitney U test) return *p-*values between 0.018 and 0.045, reflecting the fact that not only is there a consistent difference between the mutation rates of pairs of disease and carriage isolates, but also that all disease isolates have higher mutation rates than all carriage isolates (an observation that we’ve now highlighted in the manuscript, p6 line 6). For simplicity, we prefer to report the result of only a single statistical test in the manuscript, but we consider that it is important that several different approaches to testing for a difference between disease and carriage isolates leads to relatively consistent (borderline or marginally significant) *p*-values.

Independent of the precise *p-*value returned by a test, we agree with the reviewer that a sample size of four pairs is very small, and therefore that more work is required to definitively test the associations we describe. We have added this caveat to our discussion of our results (p8 lines 13-14). We have also referenced the limited power of particular comparisons at several additional points in the text (p6 lines 7-8, p9 lines 8-9, lines 22-23) and modified our language at other points (p 5 line 28, p6 lines 14-15, p8 line 3). Despite these limitations, we hope that the reviewer is satisfied that our results are sufficiently interesting to justify publication without further experiments. Our results not only favour an association between disease-association and mutation rate, but also show independent variation in overall mutation rate and mutational bias, indicate that mutational bias may be associated with both genomic base composition and longer-term pathogenic ecology, and provide evidence that deletion rates decline with genome reduction. Despite the limitations inherent in our small sample size, we hope that together, and in the context of other studies of mutation rate, genomic base composition and genome reduction, these results will be of broad interest.

Regarding claim 2. The authors assume throughout that the pathogen strain recently evolved from the commensal strain, and than a higher mutation rate evolved from a lower mutation rate. However, from the data shown, the converse scenario is equally likely. That is, the commensal strain could have evolved from the pathogen, and a lower mutation rate could have evolved from a transition to a non-pathogenic lifestyle. This possibility is never discussed in the manuscript. Perhaps the authors have data or reasons for excluding this possibility, but these reasons are not clearly stated.

1.2 We would like to thank the reviewer for raising this important point, which we agree was not properly addressed in our manuscript.

There are two related issues here: (1) the relationship between more and less pathogenic populations of *S. suis*, and (2) the relationship between disease and carriage isolates.

*Regarding (1):*

Previous studies have found evidence that the more pathogenic group of *S. suis* evolved from the less pathogenic group (Murray *et al* 2021 Mol Biol Evol; Weinert *et al*. 2015 Nature Comms). These studies analysed diversity within core genes (shared across all isolates from more and less pathogenic groups), and showed that the more pathogenic group is less genetically diverse than the less pathogenic group, and that the diversity within the more pathogenic group falls within the diversity of the less pathogenic group. We have edited the manuscript, describing this previous finding in our description of the data set (p4 lines 20-21), and also making it clearer in the discussion that this was a conclusion of previous studies (p9 lines 18-19).

While these previous results do not conclusively establish the direction of the evolution of pathogenicity, our results provide additional support for the scenario in which a more pathogenic ecology has evolved from a less pathogenic one. We find that the more pathogenic group are further from their equilibrium base composition than the less pathogenic group (p6 lines 32-34, figure S11). If mutational biases are associated with a more pathogenic ecology, the greater difference between genomic GC-content and mutational bias suggests that the ecology of the more pathogenic group is more recent than that of the less pathogenic group (we mention this in our description of these results, p7 lines 1-3).

*Regarding (2):*

We also assume in our interpretation of our results that disease isolates evolve from carriage isolates. This assumptions is based on a broader understanding of the relationship between disease and carriage isolates in opportunistic pathogens such as *S. suis*. For example, carriage is thought to be a prerequisite for both transmission to other individuals and invasive disease for *Streptococcus pneumoniae*, with carriage considered to be the first step towards invasive disease (Weiser *et al.* 2018, *Nature Reviews Microbiology*; https://doi.org/10.1038/s41579-018-0001-8). In opportunistic pathogens, such as *S. pneumoniae* and *S. suis* invasive disease is often considered an evolutionary ‘dead end’, as isolates at the sites of invasive infections are unlikely to be transmitted to new hosts.

We have edited the manuscript to make this assumption and our justification for making it clear. In particular, we have stated that it is commonly thought that in opportunistic pathogens disease isolates evolve from carriage isolates before discussing these results further in the discussion section (p8 lines 16-17), and provided a reference for this statement.

Responses to Reviewer **#**2

1.0 We would like to thank the reviewer for their positive assessment of our study and for their useful suggestions. We have provided a point by point response to comments below. Page and line numbers refer to the pdf document with highlighted changes.

**Reviewer #2:** In this paper, the authors set out to test one question, if the invasive disease isolates of the pathogen S. suis have higher mutation rate than their asymptomatic relatives? The reasoning behind this is that the living environment for the invasive disease isolates is more stressful and thus may benefit from a higher mutation rate.

To test this idea, the authors first did MA experiment in four isolates of S. suis, two invasive and two asymptomatic. 75 replicates were established for each isolate and the experiment went on for 200 days (close to 4000 generations). This experiment allows them to determine the mutation rate of these four isolates with great accuracy. And they found, in both pairs of comparison, the invasive disease isolate has significantly higher mutation rates than their asymptomatic isolate, the differences are close to two folds.

The first part of the results looks promising, but it is difficult to draw conclusion with just two pairs of comparison, the authors then did a smaller scale MA experiment in another four isolates (15 replicates each isolate, 25 days, and round 500 generations). The results from these two experiments agree with each other. Now there is significant difference in mutation rate between invasive disease isolates and asymptomatic isolates as shown by a paired t-test (P=0.03).

Apart from the major conclusion, the authors also found that there are differences in mutation spectrum between invasive disease isolates and asymptomatic isolates, and a correlation between mutational AT bias with genomic GC content.

I find the analysis in this paper robust and results supportive, and I recommend acceptance with a minor revision.

My questions are listed below:

1. Data availability

(1) The sequence reads should be made publicly available;

(2) The assembled genomes should also be made publicly available;

2.1 We have uploaded all sequence reads from the mutation accumulation experiments and the assembled genomes of the ancestral strains to NCBI. As our method of calling mutations in the evolved lines involved several approaches to exclude false variant calls within the reference-mapped assemblies of individual evolved lines, we chose not to publish these assemblies as they don’t reflect our final mutation calls. Instead we have described all mutations that we called in each line of our mutation accumulation experiments, relative to their respective reference genomes in tables S3, S8, S9, and S10. We have also provided a detailed description of our approach to excluding false calls in the methods section (p13 line 28). The accessions are now described in the main text (p15 lines 7-10). In addition, both long-read and short-read sequence reads for the assembled ancestral genomes are available on MicrobesNG and ENA websites, and the accession numbers are provided in Table S1. All data will become publicly available upon publication.

2. Sample information in the first Methods section. The authors cited another paper on the origination of the samples, it might be better to provide the information in this paper, like which part of the world are they collected and are some of them are from the same farm.

2.2 We have added information about the origins of our samples in both the Methods section (p11 lines 4-5) and in Table S1. This includes the countries of origin, and the dates of sampling. None of the isolates were sampled from the same pig farm.

3. Lines 172-173. It might be interesting to look at the spectrum of all SNV mutations (relative frequency of 6 types of SNVs). Examples like this can be found in papers that measure mutation rate.

2.3 Thank you for this suggestion. We agree that showing all six types of SNVs is likely to be of interest to readers, and may allow for easier direct comparisons with some other studies. We have added this as a supplementary figure (Figure S3) and reference it on p5 line 8. We see a consistent pattern of mutation rate elevation in disease-associated strains for all types of SNVs, with the exception of the much rarer g↔c and a↔t mutations. Although we note that there were only a small number of mutations in some of these categories (g↔c and a↔t mutations are relatively rare).

4. The scale bar of the phylogenetic tree in Fig 3 is missing.

2.4 Thank you for noticing this omission. We have now added the scale bar to this figure.

Responses to Reviewer **#**3

3.0 We would like to thank the reviewer for their constructive comments and criticisms. We have provided a point by point response to comments below. Page and line numbers refer to the pdf document with highlighted changes.

**Reviewer #3:** Despite its strengths, the initial premise of the manuscript in the abstract is incorrect because mutation rates evolve as linked traits, not as traits directly under selection. While this is a short logical walk for asexual, infrequently recombining haploids, it’s still important to clarify that selection does not “choose” genotypes on the basis of their mutation rates but rather on the side effects of these rates.

We also know a great deal about why mutation rates vary so much in the first place – it’s because of varying effective population sizes, after work by Lynch, Keightley, Foster and many others. Small population sizes enhance effects of drift relative to selection, and this weakened purifying selection allows mutations that erode replication fidelity to become frequent and even fix. So the primary hypothesis of this study should be that pathogenic lineages have smaller Ne (which seems very logical based on their newly limited lifestyle) and consequently their mutation rates increase.

3.1 We appreciate the reviewer's concern about our failure to discuss the indirect nature of selection on mutation rates. While the premise of our study does not rely on mutation rates being under direct selection, we understand that our presentation of these issues in our manuscript was brief, and could be misleading. To address this we have added discussion of the indirect nature of selection on mutation rates to our introduction (p3 lines 11-13 and 22-23), modified how we frame our study in the abstract (p2 lines 2-7), and referenced indirect selection in our discussion (p8 line 20) .

We note, however, that while selection on mutation rates will generally be indirect, some studies have found evidence that mutation rates in bacteria can also evolve under direct selection. For example, in laboratory experiments, defective DNA repair systems have been found to be directly associated with the secretion of enzymes that protect against hydrogen peroxide (e.g. Torres-Barceló et al. 2013 https://doi.org/10.1098/rspb.2013.0007). We mention this as a possible explanation of the elevated mutation rates we see in disease-associated isolates in our discussion of our results (p8 lines 21-22).

We appreciate that there is a considerable body of work that has identified an association between effective population size and mutation rate variation between species. This prediction was previously included in our introduction as the third reason that we predict that pathogens may evolve higher mutation rates. Nevertheless, many studies have identified hypermutable strains within bacterial populations (p3 lines 27-28), and have suggested that these temporary elevations in mutation rate could be beneficial, particularly for pathogens (references 17-23, p3 lines 20-22). Moreover, studies have found evidence that mutation rates may be under direct selection in bacterial populations (as discussed in the previous paragraph). While these factors may be less likely to influence long-term mutation rate evolution, we consider the extent to which these factors influence mutation rate evolution within a bacterial species an open question. Nevertheless, to address the reviewers concern we have re-ordered these predictions in our introduction, placing the hypothesis of increased drift first. We think that this better reflects the significant body of work that supports the importance of effective population size on mutation rate evolution, particularly over longer evolutionary timescales.

The primary results, higher mutation rates and spectra consistent with MMR defects (GC->AT) in pathogenic Strep. suis, make great sense. There’s limited statistical power in these comparisons unfortunately, even though the MA experiments involve a lot of well-done work that deserve celebration. There are only ~2200 mutations, or <300 per strain, and while this is a comparable amount to other well-regarded MA studies, it still makes comparisons between strains that differ in several, inconsistent ways (over relatively small evolutionary distances between them) challenging.

Still, the fact that isolates from invasive infections had somewhat (not quite twofold?) higher mutation rates than carriage isolates is intriguing, and the negative result showing no correlation between substitution rate and genome size is useful.

3.2 We thank the reviewer for their appreciation of the work that went into obtaining these results (although it still leads to rather limited statistical power). We hope that other researchers will find them useful in informing broader comparisons.

The observation of fewer deletions in smaller genomes is interesting and a novel finding as far as I know, but I’m curious how many indels you actually reliably detected? Indels are usually about ten times rarer than SNPs, meaning that the total observations may again be limiting.

3.3 We describe all the short indels we called for each strain over the course of the 200-day MA experiment in Table S9, and the long deletions we called in Table S10. Across the 50 replicate lines, we called 54 short deletions and 15 short insertions for Strain 1, 25 short deletions and 8 short insertions for Strain 2, 153 short deletions and 39 short insertions for Strain 3, and 112 short deletions and 38 short insertions for Strain 4. This leads to a relative short indel rate of between 0.08 and 0.33 indels/SNP across our four strains.

This is based on a conservative criteria for calling indels, described in our methods (p14 lines 11-17). We believe this gives us the best chance of accurately characterising variation across the four strains. Nevertheless, we accept that rates of false indel calls could still be variable across strains that have different genome sizes and genome contents, and so it remains possible that this could influence our results. We think it is more likely, however, that this variation reflects fewer genomic regions that are either prone to deletion or whose deletion is associated with little selective cost in the smaller genome strains (p7 lines 10-12), and is therefore relevant to our understanding of the process of genome reduction in bacteria.

To summarize, I really like the premise and effort of this study, and the results are valuable in general, but the causal relationships demand revision and the limited power of the cross-strain comparisons need to be acknowledged and explained.

3.3 We thank the reviewer for this positive assessment of our study and for their useful comments. We hope that they consider that our amended discussion of causal relationships (p3 lines 11-13, lines 22-23), and reflection on the limited power of our study (p6 lines 7-8, p8 lines 13-14, p9 lines 8-9, lines 22-24) is sufficient to address their concerns.
